# Prevalence and Risk Factors of Depression and Posttraumatic Stress Disorder After a Mass Shooting

**DOI:** 10.1001/jamanetworkopen.2024.2739

**Published:** 2024-03-19

**Authors:** Mohammed Abba-Aji, Angela Moreland, Salma M. Abdalla, Caitlin Rancher, Sandro Galea, Faraday Davies, Dean G. Kilpatrick

**Affiliations:** 1 School of Public Health, Boston University, Boston, Massachusetts; 2 Department of Psychiatry and Behavioral Sciences, Medical University of South Carolina, Charleston

## Abstract

This cross-sectional study examines the self-reported mental health outcomes of adults 4 years after witnessing and surviving the shooting at the Route 91 Harvest Music Festival in Las Vegas, Nevada.

## Introduction

On October 1, 2017, a lone gunman shot and killed 60 people and injured 867 others at the Route 91 Harvest Music Festival in Las Vegas, Nevada, making this mass shooting the deadliest in US history.^1^ Witnesses and survivors of mass violence incidents (MVIs) often experience depression and posttraumatic stress disorder (PTSD).^2^ However, the psychological sequelae and associated factors among witnesses and survivors of the Las Vegas MVI have yet to be examined. In this study, we documented the prevalence and risk factors of major depressive episode (MDE) and PTSD among witnesses and survivors of the Las Vegas MVI using 2021 data, 4 years after the incident.

## Methods

Participants were adult witnesses and survivors of the shooting who were recruited from a list of those who were eligible for but had not necessarily received services from the Vegas Strong Resiliency Center. Witnesses were defined as those who were present at the scene and/or sustained physical injuries, whereas survivors included family members or friends of people who were physically injured or killed. Data were collected between September 3 and November 11, 2021, using a self-administered online survey. The Boston University and Medical University of South Carolina Institutional Review Boards approved this cross-sectional study. Participants provided written informed consent. We followed the STROBE reporting guideline.

We measured MDE using a modified version of the National Women’s Study Depression module and PTSD using the National Stressful Events Survey PTSD module. Both instruments have been used previously in assessing MDE and PTSD following an MVI.^2,3^

We ran 2 separate regression models and performed bootstrap resampling with 1000 repetitions to identify the factors associated with MDE and PTSD. Two-sided *P* < .05 indicated statistical significance. All analyses were conducted from September to November 2023 using Stata SE 18 (StataCorp LLC).^4^ Data collection and analysis are summarized in the eMethods in Supplement 1.

## Results

Of the 1000 adults randomly selected from the list, 202 responded to the recruiter and 177 eligible participants completed the survey (response rate 17.7%). Participants included 132 females (74.6%) and 45 males (25.4%), with a mean (SD) age of 43.5 (12.5) years. Of these respondents, 58 (32.8%) were physically injured during the MVI and 88 (49.7%) reported having low social support. Sociodemographic characteristics of participants are reported in Table 1.

**Table 1.** Participant Characteristics

Characteristic	No. (%)
Total	177
Exposure level^a^	
Physically injured	58 (32.8)
Uninjured	113 (63.8)
Age group, y	
18-29	30 (16.9)
30-44	63 (35.6)
45-64	75 (42.4)
≥65	9 (5.1)
Sex	
Female	132 (74.6)
Male	45 (25.4)
Race and ethnicity^b^	
Other^c^	26 (14.7)
White	151 (85.3)
Educational level	
No college degree	91 (51.4)
≥College degree	86 (48.6)
Employment or work status	
Full-time	112 (63.3)
Part-time	16 (9.0)
Looking for work or unemployed	9 (5.1)
Retired	13 (7.3)
Homemaker or on parental leave	9 (5.1)
Student	13 (7.3)
On disability or sick leave	5 (2.8)
Household income, $^d^	
≤24 999	18 (10.3)
25 000-49 999	16 (9.1)
50 000-74 999	24 (13.7)
75 000-99 999	25 (14.3)
≥100 000	92 (52.6)
Social support	
Low social support	88 (49.7)
Adequate social support	89 (50.3)
Past PTE	
Sexual or physical	81 (45.8)
Other forms	61 (34.5)
None	35 (19.8)

Abbreviation: PTE, potentially traumatic event.

^a^ Six respondents were missing data for exposure category.

^b^ Race and ethnicity were self-reported in the survey. These data were collected and analyzed to understand the implication of demographic factors for PTSD and MDE outcomes and to ensure the representativeness of the study sample. The number of participants from each racial or ethnic group in the Other category was too small to analyze separately with statistical power.

^c^ Other race and ethnicity included Asian (n = 2), Black or African American (n = 1), Native Hawaiian or Pacific Islander (n = 2), and multiracial (n = 21).

^d^ Defined as the combined total annual pretax income of a household.

Eighty-seven participants (49.2%) reported past-year MDEs, while 112 (63.3%) reported past-year PTSD. Those physically injured during the MVI had a 36% higher risk of past-year MDE (adjusted risk ratio [aRR], 1.36; 95% CI, 1.01-1.84) and 32% higher risk of past-year PTSD (aRR, 1.32; 95% CI, 1.06-1.64) compared with those who were not injured. Adequate social support was associated with reduced the risk of both past-year MDE (aRR, 0.51; 95% CI, 0.35-0.74) and PTSD (aRR, 0.62; 95% CI, 0.48-0.82). Table 2 provides results of the regression analyses.

**Table 2.** Prevalence and Relative Risk for Past-Year Major Depressive Episode (MDE) and Posttraumatic Stress Disorder (PTSD) Among Survivors of Las Vegas Mass Shooting

Variable	Past-year MDE			Past-year PTSD		
	No. (%)	aRR (95% CI)	*P* value^a^	No. (%)	aRR (95% CI)	*P* value^a^
Exposure category^b^						
Physically injured	34 (58.6)	1.36 (1.01-1.84)	.047	45 (77.6)	1.32 (1.06-1.64)	.01
Uninjured	49 (43.4)	1 [Reference]		63 (55.8)	1 [Reference]	
Sex						
Male	22 (48.9)	0.94 (0.67-1.32)	.80	29 (64.4)	0.97 (0.76-1.24)	.05
Female	65 (49.2)	1 [Reference]		83 (62.9)	1 [Reference]	
Age group, y						
18-29	14 (46.7)	0.95 (0.42-2.15)	.70	23 (76.7)	1.75 (0.97-3.14)	.02
30-44	30 (47.6)	0.73 (0.32-1.67)		40 (63.5)	1.27 (0.71-2.27)	
45-64	39 (52.0)	0.87 (0.39-1.97)		44 (58.7)	1.16 (0.65-2.09)	
≥65	4 (44.4)	1 [Reference]		5 (55.6)	1 [Reference]	
Insurance coverage						
None	5 (62.5)	1.14 (0.76-1.71)	.60	6 (75.0)	1.12 (0.86-1.45)	.40
Medicare or Medicaid	11 (45.8)	0.74 (0.42-1.30)		18 (75.0)	1.07 (0.83-1.38)	
Other^c^	5 (55.6)	1.07 (0.63-1.81)		4 (44.4)	0.70 (0.37-1.33)	
Private	66 (48.5)	1 [Reference]		84 (61.8)	1 [Reference]	
Past PTE						
Sexual or physical	47 (58.0)	0.87 (0.58-1.32)	.05	58 (71.6)	1.24 (0.89-1.71)	.20
Other forms	21 (34.4)	0.58 (0.35-0.95)		34 (55.7)	1.02 (0.70-1.46)	
None	19 (54.3)	1 [Reference]		20 (57.1)	1 [Reference]	
Social support^d^						
Adequate social support	32 (36.0)	0.51 (0.35-0.74)	<.001	44 (49.4)	0.62 (0.48-0.82)	.001
Low social support	55 (62.5)	1 [Reference]		68 (77.3)	1 [Reference]	

Abbreviations: aRR, adjusted risk ratio; PTE, potentially traumatic event.

^a^ Significant at *P* = .05.

^b^ Exposure category was the main independent variable used in the logistic regression and had 2 levels: physically injured (those who sustained physical injuries during the incident) and uninjured (including those with a family or friend killed, who saw anyone else get killed or seriously injured, or who were present at the venue during the incident).

^c^ The response option in the survey was “Some other kind of health insurance.”

^d^ Social support was computed from a social support score ranging from 5 to 20, with a score of 15 or lower indicating low social support and a score over 15 indicating adequate social support.

## Discussion

We documented a high burden of MDE and PTSD among witnesses and survivors of the Las Vegas MVI. The prevalence of MDE and PTSD was considerably higher in this population than in previous studies on MVIs.^5^ Consistent with prior literature, this study showed that physical injury and low social support were associated with higher risk for MDE and PTSD.^6^

Study limitations include a small sample size, predominantly female respondents, and a low response rate, all of which may affect generalizability. Although we used bootstrapping techniques, there is a need to replicate these findings using larger sample sizes. Furthermore, various factors could have affected the mental health outcomes of the participants in the 4-year interval. This interval, however, offered a unique perspective on the long-term psychological implications of an MVI.

This study found that witnesses and survivors of the Las Vegas MVI continued to have substantial mental health challenges even 4 years later, emphasizing the need for sustained mental health support. Communities affected by MVIs should consider implementing long-term support strategies, including ongoing counseling and trauma-informed services.

## Notes

Supplement 1.
eMethods.

Supplement 2.
Data Sharing Statement
