# Supplementary material for: Prevalence and Risk Factors of Depression and Posttraumatic Stress Disorder After a Mass Shooting
Source: JAMA Netw Open. 2024 Mar 19;7(3):e242739. doi: 10.1001/jamanetworkopen.2024.2739 (PMC10951730; doi:10.1001/jamanetworkopen.2024.2739)
Supplement: Supplement 1. — eMethods. [file jamanetwopen-e242739-s001.pdf]

Supplemental Online Content

Abba-Aji M, Moreland A, Abdalla SM, et al. Prevalence and risk factors of depression and posttraumatic stress disorder after a mass shooting. *JAMA Netw Open*. 2024;7(3):e242739. doi:10.1001/jamanetworkopen.2024.2739

eMethods.

This supplemental material has been provided by the authors to give readers additional information about their work.

eMETHODS

Participants were adult victims or survivors of the October 1, 2017, Route 91 Harvest Music Festival mass shooting in Las Vegas, Nevada. These included family members or close friends of victims who were physically injured or killed at the scene. Participants were recruited in collaboration with the Vegas Strong Resiliency Center (VSRC)

© 2024 Abba-Aji M et al. *JAMA Network Open*

which maintains a list of the survivors and victims. It is important to note that that not all individuals in the center's database were actively seeking services at the time of our study. Many were simply listed as survivors or affected individuals. Data were collected between September 3 and November 11, 2021, or approximately four years after the MVI. This delay was partly due to the logistical and ethical challenges associated with engaging traumatized populations. In anticipation of conducting a larger study with these potential participants, we conducted this pilot study designed to test the most effective method of contacting and recruiting potential participants. From an initial list of 6,327 survivors and victims included on the list maintained from the VSRC with a valid email and/or mailing address, 1,000 adults were randomly selected for recruitment by Abt Associates, a survey research firm. They recruited participants using a combination of email only, mail only, and email and mail. Data were collected using a highly structured self-administered online survey. The study protocol was reviewed and approved by the Institutional Review Boards for Human Research at the first and second authors' institutions.

Of the 1,000 adults sampled in this pilot study, 202 responded to contact from Abt. Of the 202 respondents, seven partially completed the survey; two were ineligible because they were not directly affected by the shooting, and 16 refused to participate. The final sample included 177 participants who completed the survey (87.6% of those that responded to the invitation to participate).

We measured prevalence of MDE using a modified version of the National Women's Study Depression (NWSD) module and that of PTSD since the mass shooting using National Stressful Events Survey (NSES) PTSD module, both based on DSM-5 diagnostic criteria.(1) Both NWSD and NSES instruments have been previously used in assessing the prevalence of depression and PTSD, respectively, following mass traumatic events.(2–4) Our independent variables included sociodemographic characteristics (gender, age, and healthcare coverage), social support, previous exposure to PTE, and level of exposure to the mass shooting (injured vs uninjured). We selected these variables based on their established relevance in trauma research and their potential impact on mental health outcomes after a traumatic event like a mass shooting.

We ran two separate modified Poisson regression models with log link function (one for MDE and one for PTSD) to determine the factors associated with MDE and PTSD. Our analyses were restricted to fully completed responses (N=171). As a sensitivity test, we performed bootstrap resampling with 1,000 repetitions. All analyses were conducted using Stata SE version 18.(5)

eReferences

1. Kilpatrick DG, Ruggiero KJ, Acierno R, Saunders BE, Resnick HS, Best CL. Violence and risk of PTSD, major depression, substance abuse/dependence, and comorbidity: Results from the National Survey of Adolescents. J Consult Clin Psychol. 2003;71(4):692–700.

© 2024 Abba-Aji M et al. *JAMA Network Open*

2. Galea S, Ahern J, Resnick H, Kilpatrick D, Bucuvalas M, Gold J, et al. Psychological sequelae of the September 11 terrorist attacks in New York City. N Engl J Med. 2002 Mar 28;346(13):982–7.

3. Pietrzak RH, Tracy M, Galea S, Kilpatrick DG, Ruggiero KJ, Hamblen JL, et al. Resilience in the Face of Disaster: Prevalence and Longitudinal Course of Mental Disorders following Hurricane Ike. PLOS ONE. 2012 Jun 26;7(6):e38964.

4. Reuben A, Moreland A, Abdalla SM, Cohen GH, Friedman MJ, Galea S, et al. Prevalence of Depression and Posttraumatic Stress Disorder in Flint, Michigan, 5 Years After the Onset of the Water Crisis. JAMA Netw Open. 2022 Sep 20;5(9):e2232556.

5. StataCorp. 2023. Stata Statistical Software: Release 18. College Station, TX: StataCorp LLC.

© 2024 Abba-Aji M et al. *JAMA Network Open*
